# Fast quantitative MRI using controlled saturation magnetization transfer

**DOI:** 10.1002/mrm.27442

**Published:** 2018-09-14

**Authors:** Rui Pedro A.G. Teixeira, Shaihan J. Malik, Joseph V. Hajnal

**Affiliations:** ^1^ School of Biomedical Engineering and Imaging Sciences King’s College London London United Kingdom; ^2^ Centre for the Developing Brain King’s College London London United Kingdom

**Keywords:** DESPOT, JSR, MT, relaxometry, steady‐state, VFA

## Abstract

**Purpose:**

This study demonstrates magnetization transfer (MT) effects directly affect relaxometry measurements and develops a framework that allows single‐pool models to be valid in 2‐pool MT systems.

**Methods:**

A theoretical framework is developed in which a 2‐pool MT system effectively behaves as a single‐pool if the RMS RF magnetic field ($B_{1}^{\mathrm{rms}}${\text{B}}_{1}^{{{\text{rms}}}}) is kept fixed across all measurements. A practical method for achieving controlled saturation magnetization transfer (CSMT) using multiband RF pulses is proposed. Numerical, Phantom, and in vivo validations were performed directly comparing steady state (SS) estimation approaches that under correct single‐pool assumptions would be expected to vary in precision but not accuracy.

**Results:**

Numerical simulations predict single‐pool estimates obtained from MT model generated data are not consistent for different SS estimation methods, and a systematic underestimation of T_2_ is expected. Neither effect occurs under the proposed CSMT approach. Both phantom and in vivo experiments corroborate the numerical predictions. Experimental data highlights that even when using the same relaxometry method, different estimates are obtained depending on which combination of flip angles (FAs) and TRs are used if the CSMT approach is not used. Using CSMT, stable measurements of both T_1_ and T_2_ are obtained. The measured T_1_ ${(T}_{1}^{\mathrm{CSMT}})$) depends on $B_{1}^{\mathrm{rms}}${\text{B}}_{1}^{{{\text{rms}}}}, which is therefore an important parameter to specify.

**Conclusion:**

This work demonstrates that conventional single pool relaxometry, which is highly efficient for human studies, results in unreliable parameter estimates in biological tissues because of MT effects. The proposed CSMT framework is shown to allow single‐pool assumptions to be valid, enabling reliable and efficient quantitative imaging to be performed.

## 1 INTRODUCTION

MRI is routinely used as a highly sensitive soft‐tissue imaging modality invaluable for clinical diagnosis. MR images are qualitative, in that the voxel intensity values are related to underlying tissue properties, but are also dependent on specific details of scanner hardware and software^1^ and on parameter settings that are often uniquely optimized by each imaging center. These tissue‐weighted images are familiar to the radiologists who interpret them, however, lack of consistency hampers automated analysis and can impede group or longitudinal comparisons. In a world of Big Data^2^ there is an increased need to move toward quantitative MRI (qMRI), where image values are direct measurements of relevant tissue properties.^3^, ^4^, ^5^, ^6^ Such an approach may allow more accurate tracking of disease progress, improve characterization of global population variances, and provide statistical power for examining rare diseases.^4^ A challenge for qMRI is the highly complex nature of biological tissue—in a typical voxel of an MR image, tissue can be well described by mobile component(s), or free‐pool(s) of protons (eg, liquid water), in close contact with a less mobile, proton‐rich, macromolecular matrix^7^, ^8^ (restricted‐pool[s] of protons). Although macromolecular constituents do not necessarily generate measurable signal because of their short spin‐spin relaxation times ($T_{2}=1/R_{2}$), they do interact and influence the imaging experiment^7^, ^8^, ^9^, ^10^, ^11^, ^12^, ^13^, ^14^, ^15^, ^16^, ^17^ as their proton density ($M_{0}$) and spin‐lattice recovery time ($T_{1}=1/R_{1}$) are of comparable magnitude to visible components. Extensive literature has explored interaction between these pools, which is often referred to as magnetization transfer (MT).^9^, ^18^, ^19^

Extended data acquisitions are required to obtain sufficiently diverse measurements to fit the plethora of parameters involved in full multi‐pool models of tissue and this has limited their adoption in larger scale studies and in the clinic. A much more common strategy has been to use single‐pool models, in which signals from tissue at each imaged location are treated as if they may be characterized by a single set of parameters $M_{0}$ or proton density, $T_{1}$ and $T_{2}$.^4^, ^5^, ^20^, ^21^ Use of a simplified model along with highly efficient acquisitions results in a capability for producing whole brain high resolution tissue parameter maps in clinically relevant scan durations. Prominent methods include driven equilibrium single pool observation of T_1/2_ (DESPOT),^20^, ^22^ MP2RAGE,^23^ magnetic resonance fingerprinting (MRF),^5^ and others.^4^, ^24^, ^25^, ^26^ These methods can all provide robust results, but articles featuring different methods and/or from different centers have reported a wide range of tissue parameter values.^27^, ^28^ For example, recent work from Bojorquez et al.^28^ reveals T_1_ values for healthy white matter (WM) at 3 T range from 699‐1735 ms, which is much larger than the estimated parameter uncertainty for individual studies. T_2_ values measured by these methods are also routinely reported as lower than expected.^29^, ^30^ We hypothesize that this disparity of results stems directly from the fact that standard single‐pool models do not account for MT effects, leading to systematic errors in the resulting parameter estimation. The proposed controlled saturation magnetization transfer (CSMT) framework directly addresses this issue and puts forward the tools to enforce 2‐pool MT systems to precisely follow single‐pool behavior. Building on quantitative MT literature, we propose to augment conventional M_0_ and T_1_ definitions to be defined by MT rather than being hindered by it. We achieve this by observing that for short TR steady state (SS) methods, 2‐pool MT systems behave as a single‐pool with an apparent M_0_ and T_1_ that depends on the average RF power level (here parameterized via the RMS RF magnetic field (B_1_): $B_{1}^{\mathrm{rms}}$),^31^, ^32^ and a 2‐pool system can be forced to have consistent single pool M_0_ and T_1_ across measurements if $B_{1}^{\mathrm{rms}}$ is held fixed. Under this newly defined sampling regime, the parameters M_0_ and T_1_ derived from single‐pool relaxometry become explicitly dependent on $B_{1}^{\mathrm{rms}}$, but may then be reliably reported along with the associated $B_{1}^{\mathrm{rms}}$. This paper articulates and explores this framework and presents evidence to confirm that consistent and stable quantitative results can be achieved, paving the way for fast reliable relaxometry that may be suitable for both clinical and research use.

## 2 THEORY

### 2.1 2‐pool system, single pool model

**Figure 1:**
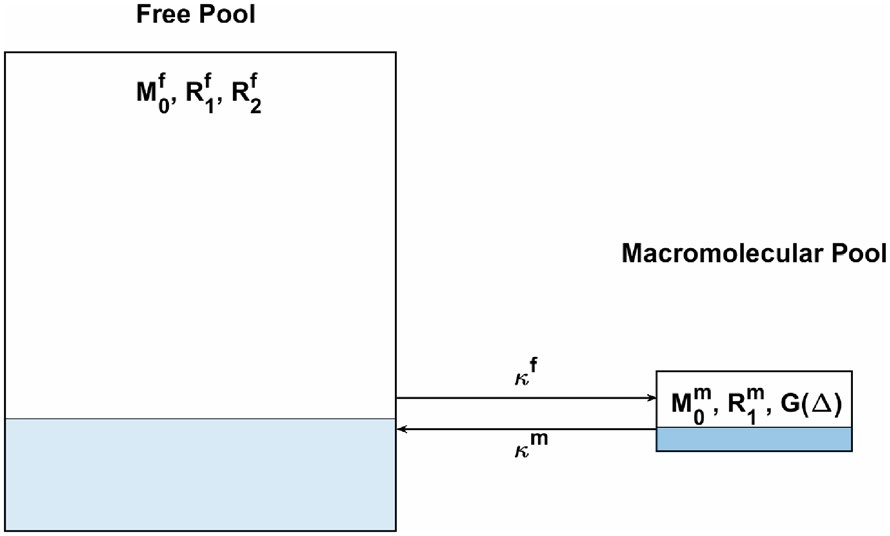
Schematic representation of 2‐pool system. Both free pool (left) and macromolecular pool (right) have their own equilibrium magnetization (M_0_) spin‐lattice interaction rate (R_1_). Free pool has an intrinsic spin‐spin interaction rate R_2_. Macromolecular spin‐spin interaction is parameterized via the absorption line shape G. Both pools can exchange at a first order exchange rate

The ability to obtain whole‐brain 3D quantitative images with 1 mm isotropic resolution in a clinically feasible time (~10 min),^20^, ^21^, ^29^, ^33^ has been enabled by the use of short TR sequences. The main assumption of the CSMT framework is that the mean macromolecular saturation effect < W> that occurs at each excitation pulse,^31^, ^34^ can be approximated by a continuous wave equivalent $\leq \overset{¯}{W}>\;$ that assumes an average saturation occurring throughout the entire imaging acquisition. A similar approach has been previously used to compare macromolecular saturation of different pulsed strategies based on a RF duty‐cycle.^31^, ^32^, ^35^ Here, we chose to parameterize $\overset{¯}{W}>$ via $B_{1}^{\mathrm{rms}}$ as it is commonly reported across different vendors as an SAR‐specific metric. The current work proposes that, for SS sequences where a dynamic equilibrium is established and the longitudinal magnetization for each pool returns to the same value at intervals of TR, a relationship between the background pool‐induced saturation and the observed recovery of the free pool magnetization (such that it is driven by newly defined parameters $M_{0}^{\mathrm{CSMT}}$ and $T_{1}^{\mathrm{CSMT}}$) can be established. To achieve this, we build on the Bloch‐McConnell equation^36^ under MT assumptions^8^:

(1) dMxfdt=-R2Mxf+Δω0Myf-ImγB1Mzf,

(2) dMyfdt=-Δω0Mxf-R2Myf+ReγB1Mzf

(3) dMzfdt=ImγB1Mxf-ReγB1Myf+R1fM0f-Mzf-κfMzf+κmMzmand

(4) $$\frac{dM_{z}^{m}}{\mathrm{dt}}=\kappa^{f}M_{z}^{f}+R_{1}^{m}\left(M_{0}^{m}-M_{z}^{m}\right)-\kappa^{m}M_{z}^{m}-\leq W>M_{z}^{m}$$

where ${\Delta \omega }_{0}$ is the off‐resonance frequency shift of the static polarizing magnetic field, γ is the gyromagnetic ratio, and B_1_ is magnitude of the RF magnetic field. The superscripts f and m identify, respectively, the free and macromolecular pools of magnetization, *k^m^* and $\kappa^{f}$ are first order exchange rates between the 2 pools (Figure 1).

For a pulsed experiment with short TR, we postulate that the recovery amount of macromolecular longitudinal recovery is limited and hence, the effect of the instantaneous mean saturation rate can be approximated as a continuous wave irradiation equivalent^31^, ^32^ such that < *W*> is decoupled from specific details of the irradiation field B_1_ and is substituted by the average saturation per TR: $\overset{¯}{W}=\pi {\left({\gamma B}_{1}^{\mathrm{rms}}\right)}^{2}G\left(\Delta \right)$. Here, $B_{1}^{\mathrm{rms}}$ represents the RMS amplitude of an arbitrary RF pulse whose energy is spread over a repetition period TR, and G is the absorption line shape of the macromolecular pool dependent on its spin‐spin relaxation rate, $R_{2}^{m}$, and parameterized by the frequency offset δ. $R_{2}^{m}$ is large enough to ensure that RF pulses purely saturate the macromolecular pool (and hence $R_{2}^{m}$ is omitted from Equations (1)‐(4) and Figure 1). G is commonly accepted to have broad frequency response^34^, ^37^ and hence can be approximated as constant for a wide range of values of δ. In this mathematical description, we refer to relaxation rate R_1_, which is the reciprocal of the relaxation time T_1_, as this notation occurs more naturally in the equations and improves readability. As we assume $\overset{¯}{W}$ decoupled from the irradiation field, algebraic manipulation of Equations (1A)‐(1C), summarized in Appendix A, lead to identification of a $B_{1}^{\mathrm{rms}}$‐dependent relaxation rate

(5) $$R_{1}^{\mathrm{CSMT}}=R_{1}^{f}+\kappa^{f}\left(1-\frac{\kappa^{m}}{R_{1}^{m}+\kappa^{m}+\leq \overset{¯}{W}>}\right)$$

and equilibrium magnetization.

(6) $$M_{0}^{\mathrm{CSMT}}=\frac{1}{R_{1}^{\mathrm{CSMT}}}\left[-\frac{\kappa^{m}\frac{dM_{z}^{m}}{\mathrm{dt}}}{R_{1}^{m}+\kappa^{m}+\overset{¯}{W}}+\frac{R_{1}^{m}M_{0}^{m}}{R_{1}^{m}+\kappa^{m}+\leq \overset{¯}{W}>}+R_{1}^{f}M_{0}^{f}\right]$$

that suffice to describe the time evolution of the 2‐pool system. In the limit where $B_{1}^{\mathrm{rms}}\to \infty$ the known solution for a fully saturated system^38^ is recovered.

(7) $$R_{1}^{\mathrm{CSMT}}\left(B_{1}^{\mathrm{rms}}\to \infty \right)=R_{1}^{f}+\kappa^{f}$$

(8) $$M_{0}^{\mathrm{CSMT}}\left(B_{1}^{\mathrm{rms}}\to \infty \right)=\frac{R_{1}^{f}M_{0}^{f}}{R_{1}^{f}+\kappa^{f}}$$

This demonstrates that it is possible to describe a 2‐pool MT system as a single‐pool if $\frac{dM_{z}^{m}}{\mathrm{dt}}$ can be considered constant. Such is the case for short TR SS conditions where, for a repetition period, $\frac{dM_{z}^{m}}{\mathrm{dt}}\approx 0$. Therefore, the 2‐pool system can be characterized as a single pool with the definition of proton density and spin‐lattice interaction extended to $M_{0}^{\mathrm{CSMT}}$ and $R_{1}^{\mathrm{CSMT}}$.

### 2.2 CSMT

**Figure 2:**
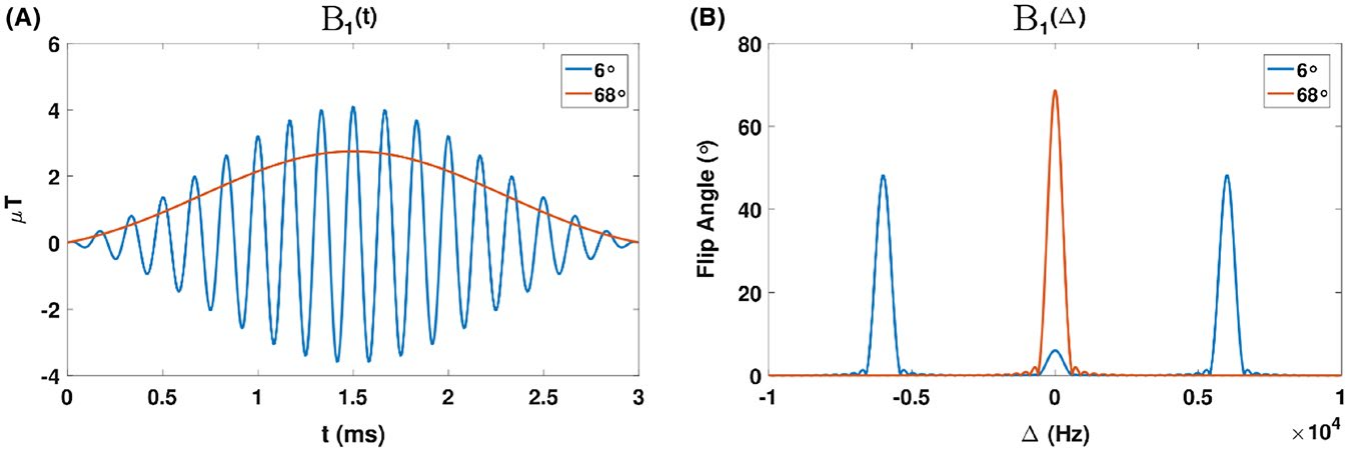
Time (A) and frequency (B) representation of RF waveforms with $\alpha^{f}=\alpha^{\mathrm{ref}}=68^{\circ }$ (red) and $\alpha^{f}=6^{\circ }$ (blue). By construction, both pulses have the same energy. Different target FAs can be achieved by balancing RF energy between central and outer bands—the maximum${\;\alpha }^{f}$ for this design is $\alpha^{\mathrm{ref}}=$ 68° and the 2 outer bands are 0 at this point

Typically, to enhance sensitivity of the relaxometry methods to the parameters of interest, a finely tuned combination of flip angles (FAs) and TR are selected^29^, ^39^, ^40^ without consideration of the resulting $B_{1}^{\mathrm{rms}}$. Equations (2A) and (2B) imply that single‐pool relaxometry methods can be sufficient to precisely perform qMRI on 2‐pool systems with MT, provided the RF saturation conditions are held constant across any necesssary variations in FA and TR Hence, we seek a pulse design strategy that allows free choice of free‐pool rotation angle, $\alpha^{f}$, and TR while keeping the total macromolecular pool saturation^31^, ^34^, ^37^ averaged over a fixed TR period $\leq \overset{¯}{W}>\;$ constant. This can be achieved because $\alpha^{f}$ is proportional to the time integral of the excitation field B_1_ whereas $\;{\left({\gamma B}_{1}^{\mathrm{rms}}\right)}^{2}=\frac{1}{\mathrm{TR}}\int_{0}^{\tau_{\mathrm{RF}}}\gamma^{2}B_{1}^{2}\left(t\right)dt$ is an integral of $B_{1}^{2}$ evaluated over the pulse duration $\tau_{\mathrm{RF}}$. Our solution is a non‐selective multi‐band pulse (Figure 2), which is a variant of a strategy more commonly adopted for simultaneous multi‐slice imaging applications.^41^, ^42^ A 3‐band pulse waveform is used, with the central band used to excite the free pool magnetization and the other 2 bands at offset frequencies ±Δ used solely to induce saturation of the macromolecular pool. Assuming the absorption spectrum of the macromolecular pool is sufficiently broad (ie, $G\left(\pm \Delta \right)=G\left(0\right)$), the total saturation $\leq \overset{¯}{W}>\;$ is held constant as long as the energy of this pulse ($\int_{0}^{\tau_{\mathrm{RF}}}\gamma^{2}B_{1}^{2}\left(t\right)dt\;)$ does not change. The free pool FA is set by changing the magnitude of the central band, with the outer bands adjusted to keep pulse energy equal to a reference rotation $\alpha^{\mathrm{ref}}$ parameterized via $B_{1}^{\mathrm{ref}}$

(9) $$B_{1}^{\mathrm{CSMT}}\left(t\right)\;=\;\left[\frac{\alpha^{f}}{\alpha^{\mathrm{ref}}}B_{1}^{\mathrm{ref}}\left(t\right)+\kappa B_{1}^{\mathrm{ref}}\left(t\right)\cos\left(2\pi \Delta t\right)\right],\;s.t.\int_{0}^{\tau_{\mathrm{RF}}}{\left(\gamma B_{1}^{\mathrm{CSMT}}\left(t\right)\right)}^{2}dt=\int_{0}^{\tau_{\mathrm{RF}}}{\left(\gamma B_{1}^{\mathrm{ref}}\left(t\right)\right)}^{2}dt$$

where κ is chosen to satisfy the pulse energy constraint. An illustrative example can be seen in Figure 2.

Therefore, a single RF waveform affords simultaneous control of restricted pool saturation as well flexibility to set the desired rotation of the free pool of protons. This is distinct from the conventional approach to induce saturation of the restricted pool of protons in clinical systems that interleaves off‐resonance saturation pulses with on‐resonance imaging pulses.^8^, ^10^, ^14^

## 3 METHODS

For single‐pool relaxometry to be a useful approach, the estimated parameters should be independent of the choice of measurements made and the fitting procedure used. In the previous sections, we have presented the theory that enables this in 2‐pool MT systems if $B_{1}^{\mathrm{rms}}$ is held fixed across measurements. This is not the case for conventional MRI, where each measurement will effectively evolve with a different recovery rate that will depend on both tissue properties and measurement$B_{1}^{\mathrm{rms}}$, but single pool estimation is enabled through the proposed CSMT framework. To test this prediction, numerical, phantom, and in vivo testing were performed based on SS data using spoiled gradient echo (SPGR) and balanced steady state‐free precession (bSSFP) sequences with a range of excitation FA ($\alpha_{\mathrm{SPGR}}^{f}=6^{\circ },\;8^{\circ },\;10^{\circ },\;12^{\circ },\;14^{\circ },\;16^{\circ }$, $\alpha_{\mathrm{bSSFP}}^{f}=15^{\circ },\;25^{\circ },\;35^{\circ },\;45^{\circ },\;55^{\circ },\;65^{\circ },\;25^{\circ },\;55^{\circ }$ with a respective RF phase cycling increment $\Phi_{\mathrm{Incr}}=\;\pi ,\;\pi ,\;\pi ,\;\pi ,\;\pi ,\;\pi ,\;0,\;0\;\;\mathrm{rad}$). A fixed TR of 7 ms was used for all sequences. The resulting data were fitted using both the well‐known driven equilibrium single pulse observation of T_1_/T_2_ (DESPOT1/2)^20^ method and the recently proposed joint system relaxometry.^29^ Both methods make use of the same single‐pool model; DESPOT first estimates M_0_ and T_1_ from SPGR data and makes use of the estimated T_1_ to estimate a second M_0_ and T_2_ from a bSSFP signal model. The joint system relaxometry (JSR) approach achieves the same outcome in a single estimation step. These methods are expected to differ only in precision while maintaining accuracy,^29^ provided the homogeneous single‐pool assumption is valid but will result in different parameter estimations otherwise. DESPOT1 and DESPOT2 approaches made use of magnitude data of both SPGR and bSSFP signals, whereas JSR used real and imaginary channels of the bSSFP signal. Parameter estimates of each noise‐independent trial are obtained based on a least‐squares criterion using MATLAB 2016b (The MathWorks, Natick, MA) *lsqnonlin* routine. The objective function was defined as the sum of the squared differences between the single‐pool model and the simulated signal intensity. The stopping criteria were a tolerance of 10^−15^ on the cost function value or a maximum of 500 iterations. DESPOT1 fitting used the conventional approach of linearization^20^ of the Ernst signal, so a simple linear least squares estimate for T_1_ was obtained in this case only. A C++ module integrated within MRtrix (https://www.mrtrix.org/) software is currently under development and will be made publicly available on https://github.com/mriphysics.

### 3.1 Numeric validation

Monte Carlo simulations of 2‐pool pulsed MT model systems were performed based on Equations (B9) and (B11) of Appendix B where a matrix formalism^34^, ^37^ is used to describe bSSFP and SPGR signals, respectively. Note that ground truth 2‐pool data were simulated assuming pulsed saturation at each excitation (as per Equation (B3)) followed by periods of free recovery (as per Equation (B1)). Estimated single‐pool values are compared to the expected $T_{1}^{\mathrm{CSMT}}$ obtained from the CW approximation and ground‐truth T_2_. Both non‐CSMT and CSMT (reference FA of $\alpha^{\mathrm{ref}}=65^{\circ }$) conditions were studied, each with RF durations of $\tau_{\mathrm{RF}}=0.614\;\mathrm{ms}$. For non‐CSMT, $B_{1}^{\mathrm{rms}}$ is calculated from the specified free‐pool FA $\alpha^{f}$, whereas under CSMT, $B_{1}^{\mathrm{rms}}$ was kept fixed throughout all measurements. For each independent instance, Gaussian distributed noise with zero mean and SD 0.002 $M_{0}^{f}$ was added to both the real and imaginary components of the simulated signal. Tissue parameters were extracted from Gloor et al.^34^

(10) $$M_{0}^{f}=1;\;M_{0}^{m}=0.157;\;k^{f}=4.45s^{-1};\;k^{m}=28.34s^{-1};\;R_{1}^{f}=1.1s^{-1};\;R_{1}^{m}=1s^{-1};\;R_{2}^{f}=12.3s^{-1};\;G=14\mu s$$

To explore the robustness and limits of the CW approximation, a comparison between pulsed and non‐pulsed signal models and bSSFP steady‐state simulations was performed using both approaches and summarized in Supporting Information Figure S1.

### 3.2 Experimental validation

All measurements were defined as 3D sagittal acquisitions with a FOV of 250 × 250 × 250 mm at 0.8 mm isotropic resolution. Sampling bandwidth was kept at 959 Hz/pixel and SENSE acceleration factors of 2 were applied in both anterior–posterior and right–left directions. Data were sampled using both standard RF pulses, for which RF power varies with FA (non‐CSMT), and the proposed CSMT pulses, where RF power is held constant. Correct transmit field knowledge was obtained via the AFI approach^43^, ^44^ with TR_1_/TR_2_ = 40/200 ms with maximum allowed gradient spoiling between each TR. The AFI nominal FA was set to 80°. The FOV was kept the same as for the SPGR and bSSFP measurements, but the acquired voxel size was set at 4.46 mm isotropic. The acquisition time was 2 min 15 s per SPGR/bSSFP volume and 1 min 46 s for the AFI field map.

Phantom validation was obtained on a spherical container filled with hair conditioner (TRESemme, Unilever PLC, London, UK) that is known to exhibit substantial MT effects.^45^ For both non‐CSMT and CSMT conditions, DESPOT1, DESPOT2, and JSR estimates were generated by model fitting to all the data in a circular region in the center of the phantom where B_0_ and B_1_ inhomogeneities are mitigated. To further explore potential inconsistencies, different subsets of the measured FAs were selected, and parameters were estimated for each data subset based on the JSR approach.

In vivo validation sought to corroborate our phantom experiment. Hence, the same FA measurements were performed on 3 different healthy volunteers who each gave written, informed consent according to local ethics requirements. Furthermore, we obtained data to validate the prediction that T_1_ varies as a function of $B_{1}^{\mathrm{rms}}$. To achieve this, we used the CSMT approach to sample SPGR data $\left(\alpha_{\mathrm{SPGR}}^{f}=6^{\circ },\;12^{\circ },\;18^{\circ },\;\mathrm{TR}=15\mathrm{ms},\;\mathrm{Acq}.\mathrm{Res}=1.2\times 1.2\times 1.2{\mathrm{mm}}^{3}\right)$for different $B_{1}^{\mathrm{rms}}$ which vary between 0.2 μT and 2.0 μT in increments of 0.2 μT on a single volunteer. T_1_ maps were then obtained based on the DESPOT1 method.

WM and gray matter (GM) masks were obtained for each subject's data using the FSL5.0 FAST tool (www.fsl.fmrib.ox.ac.uk) operating on the SPGR image with highest T_1_ contrast. The obtained masks were eroded based on a spherical element, radius 6 voxels, to create subject‐specific anatomic regions of interest (ROI) of ~2000 voxels, which were used for a summary comparison of parameter estimates for all FA subsets across all subjects.

## 4 RESULTS

### 4.1 Numerical validation

**Figure 3:**
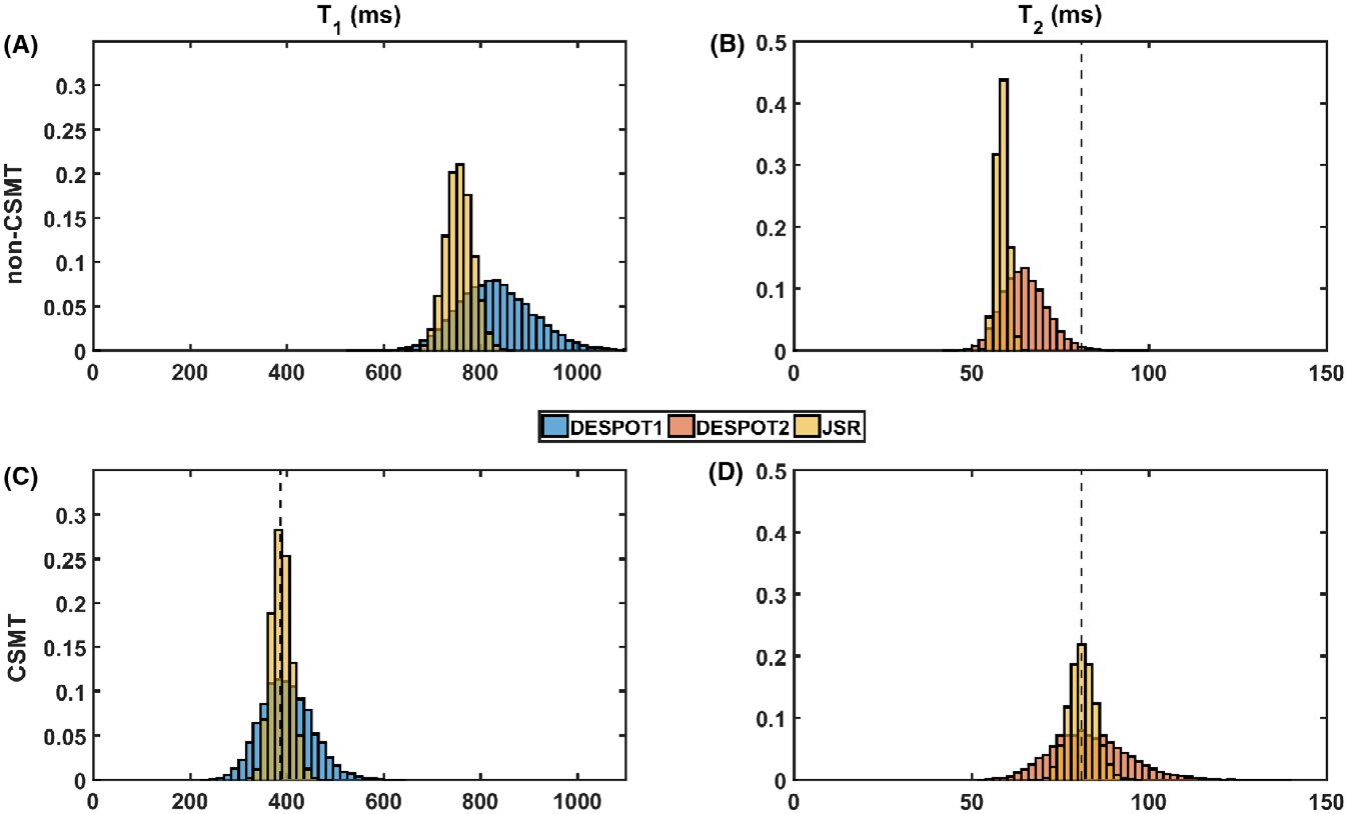
Summary comparison of MC simulations from pulsed saturation ground truth for both non‐CSMT (A and B) and CSMT sampling conditions (C and D). Correct T_2_ and expected T_1_^CSMT^ are highlighted by dashed black lines

Direct comparison between DESPOT1/2 and JSR single‐pool estimates of T_1_ and T_2_ obtained from the Monte Carlo simulation of 2‐pool pulsed MT data are summarized in Figure 3.

Figure 3A highlights an inconsistency between DESPOT and JSR estimates under non‐CSMT conditions and Figure 3B demonstrates that both analyses methods systematically underestimate the true T_2_ value (dotted vertical line). Both effects are removed under CSMT conditions (Figure 3C and D).

### 4.2 Experimental phantom validation

**Figure 4:**
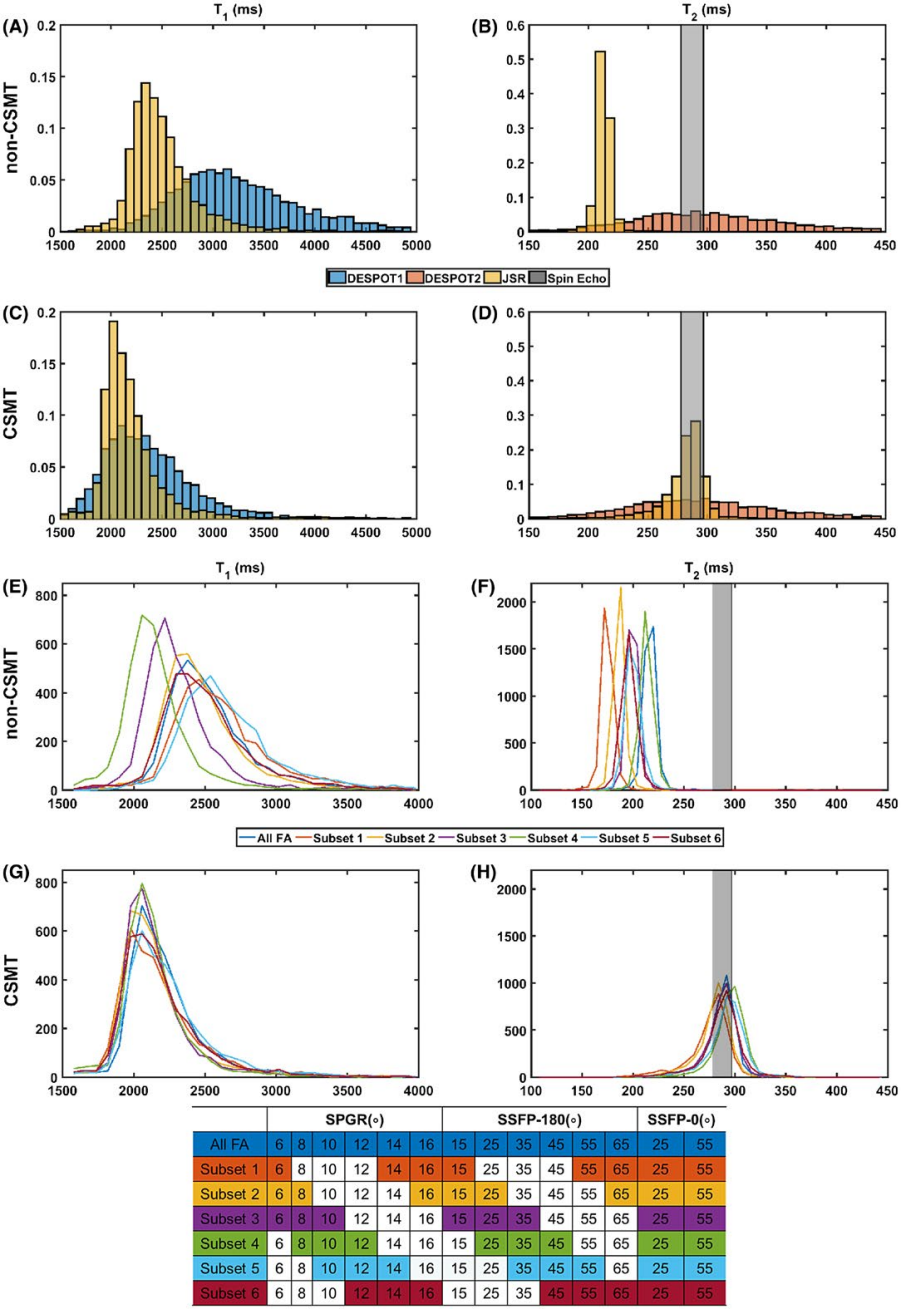
Experimental validation of using CSMT framework to induce constant MT saturation throughout SS measurements. All histograms represent single‐pool estimated parameters from a circular ROI in the centre of the spherical phantom. Subplots (A), (B), (E), and (F) correspond to histograms obtained from data sampled under conventional non‐CSMT framework, whereas subplots (C), (D), (G), and (H) demonstrate histograms sampled under the proposed CSMT framework. Subplots (A)–(D) compare distributions where all measured data was used to estimate relaxometry parameters based on DESPOT1, DESPOT2, and JSR approaches. Subsets of the measured data, summarized in the table at the bottom, were used to estimate relaxometry parameters using JSR approach—subplot (E) to (F). The reader is invited to note that when using different estimation approaches or using different measurements to obtain relaxometry estimates of single‐pool system, only precision, and not accuracy, is expected to vary. This condition is not satisfied under conventional imagining conditions. All histograms agree independently of the approach used to estimate the relaxometry parameters once CSMT is used

For both non‐CSMT and CSMT conditions, DESPOT1 (blue), DESPOT2 (orange), and JSR (yellow) estimates were generated by model fitting to all the data (Figure 4A‐D).

From Figure 4A and B, it can immediately be seen that under conventional (non‐CSMT) conditions, DESPOT and JSR estimation do not agree. As expected from our mathematical derivation and numerical validation, once CSMT is used to ensure constant saturation of the macromolecular pool (Figure 4C and D), the data behaves according to a single‐pool model across measurements, and the relaxation parameters estimated using the 2 methods vary only in precision (spread of obtained distributions) but not in the average final value obtained (accuracy). Furthermore, T_2_ estimations (Figure 4D) are consistent with multi‐echo spin‐echo data, which is indicated by gray bars in Figure 4.

To further demonstrate this effect, subsets of the measured FAs were selected, and parameters were estimated for each subset based on the JSR approach. A comparison of the different distributions obtained can be seen in Figure 4E‐H. For a true single‐pool system, using different permutations of measurements to estimate parameters is expected to vary precision of estimation but not accuracy. Under non‐CSMT conditions (Figure 4E and F), the estimated T_1_ and T_2_ are clearly observed to vary with the subset of data used, with T_2_ consistently underestimated. With CSMT acquisition (Figure 4G and H), both effects disappear, as all the histograms obtained for different subsets align on top of each other. This simple experiment corroborates our hypothesis that a 2‐pool system effectively behaves as a single‐pool once constant saturation of MT is guaranteed.

### 4.3 Experimental in vivo validation

**Figure 5:**
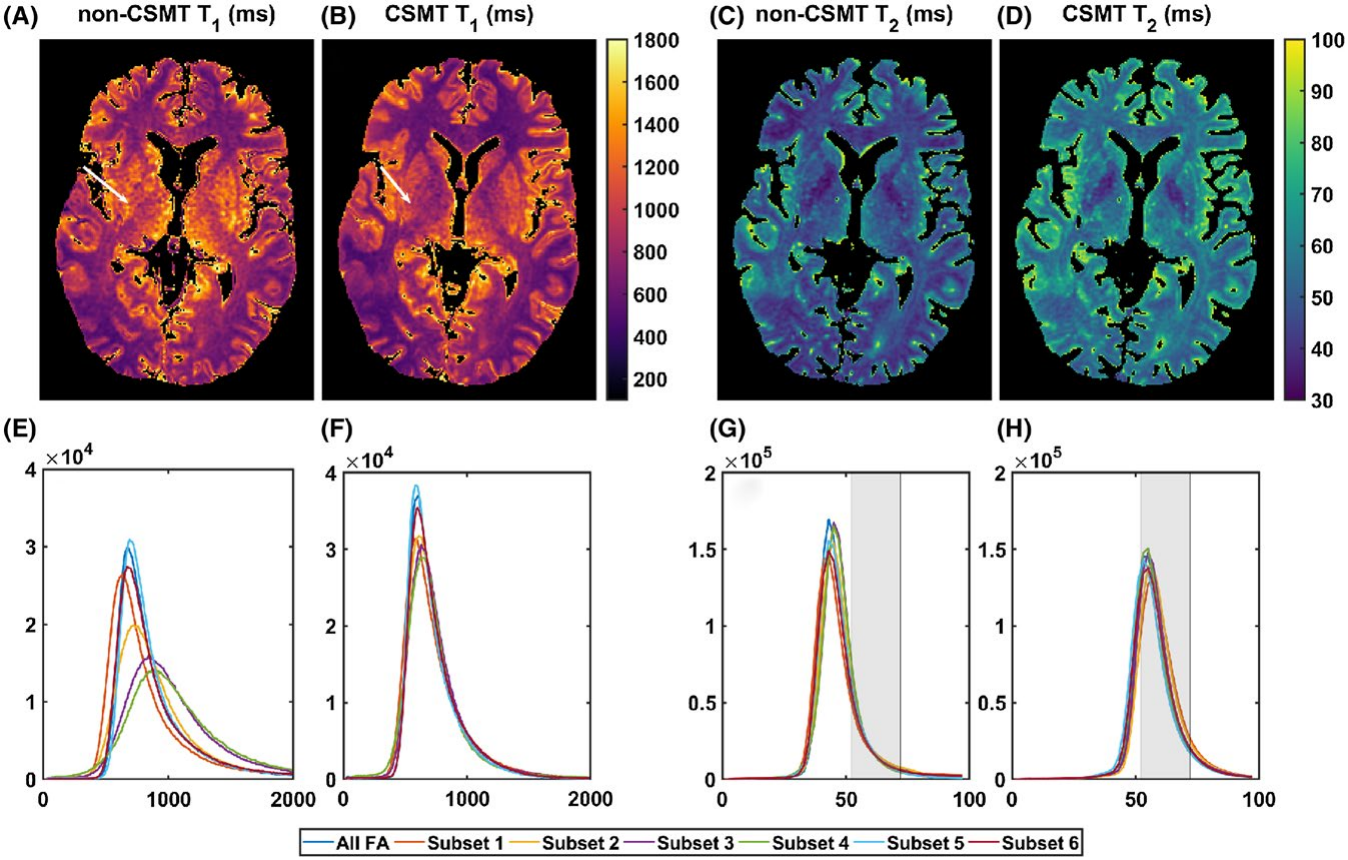
(A–D) Representative T_1_ and T_2_ maps under non‐CSMT and CSMT conditions using all FA subsets. (E–H) Top: respective whole brain WM subset analysis as per Figure 4. Bottom: box and whiskers plots of respective whole brain WM T_1_ and T_2_ distributions. Gray bars in (G) and (H) highlight the range of spin‐echo values summarized in previous literature^46^ at the same field strength

The top row of Figure 5 shows direct comparison between estimated JSR parameter maps obtained under non‐CSMT and CSMT conditions for a single volunteer. Sampling under constant RF power results in clearer depiction of anatomically distinct regions, particularly in deep GM (Figure 5A and B, arrows), suggesting that estimation noise is reduced overall, corroborating our hypothesis that the measured data are more consistent with a single‐pool model. T_2_ values under CSMT are in agreement with reported literature gold‐standard spin‐echo‐based values at the same field strength.^46^ The subset analysis of Figure 4E‐H was performed and is summarized in Figure 5E‐H. As with the phantom experiment, the conventional measurement approach results in variation in the average estimated relaxation times that is dependent on which subsample of measurements is used to estimate the single‐pool model. Under CSMT conditions, estimated relaxation times are independent of the FAs chosen.

**Figure 6:**
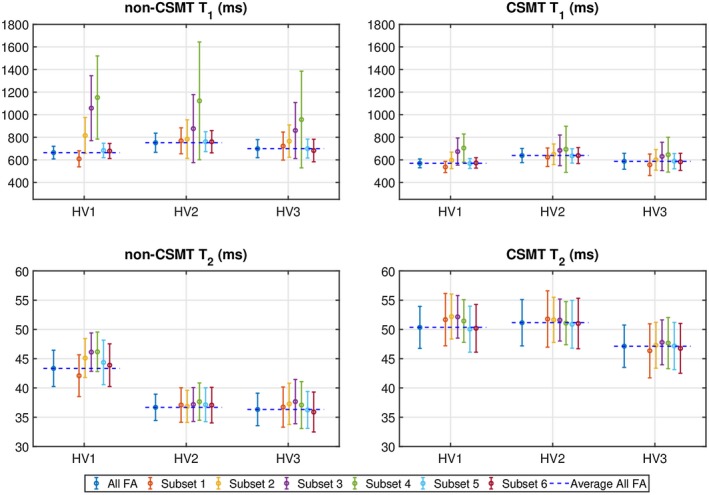
WM ROI analysis across all different subjects and subsets. Horizontal dashed blue lines indicate mean T_1_ and T_2_ values obtained when using all measurements available for the non‐CSMT and CSMT conditions

Figure 6 shows a summary comparison of obtained T_1_ and T_2 _WM mean and SDs for all subsets in all volunteers. It is evident that some subsets are more suitable than others for estimating T_1_. However, for each subset, the spread of T_1_ values is much reduced when CSMT conditions are used as highlighted by the systematically lower variances of T_1_ estimation even when a poor choice of FAs is used (subsets 3 and 4). Furthermore, for both T_1_ and T_2_, the results are more consistent across subsets as the observed RMS difference between the superset (all measurements are used) and each subset is always lower under CSMT conditions as summarized in Table 1.

**Table 1:** RMS difference between the superset of FA considered as reference and each estimated subset from Figure 6 for each HV

	***T_1_*** ***(ms)***		***T_2_*** ***(ms)***	
	***non‐CSMT***	***CSMT***	***non‐CSMT***	***CSMT***
HV1	648	176	4.67	3.12
HV2	393	74	1.30	0.97
HV3	312	80	1.91	1.22

Abbreviations: HV, healthy volunteer.

Under CSMT conditions all subsets are in better agreement with the estimated value using all available data.

**Figure 7:**
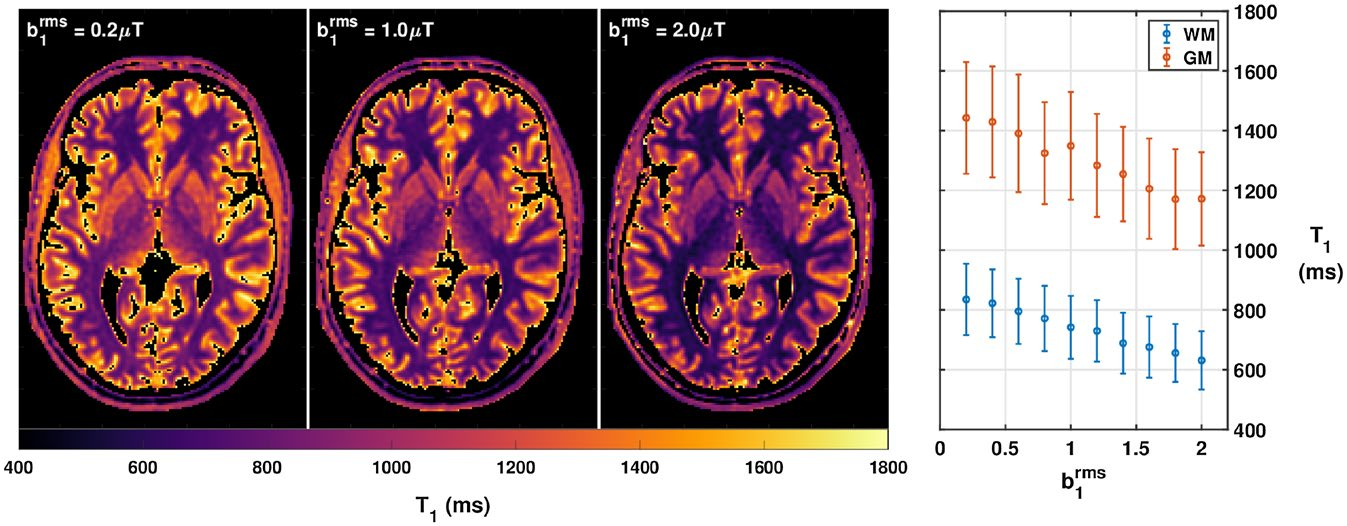
Direct comparison of T_1_ maps obtained at different $B_{1}^{\mathrm{rms}}$ using otherwise identical acquisition parameters. Rightmost plot summarizes the variation (mean ± SD) of estimated T_1_ obtained at different $B_{1}^{\mathrm{rms}}$ in WM and GM using a tissue class whole brain segmentation

Furthermore, we seek to validate our theoretical description where both M_0_ and T_1_ recovery parameters are expected to vary with $B_{1}^{\mathrm{rms}}$. This is experimentally highlighted in Figure 7, which shows T_1_ maps obtained from measurements at different constant values of $B_{1}^{\mathrm{rms}}$, demonstrating a clear change in this estimated parameter. The same effect was observed on M_0_ estimation.

## 5 DISCUSSION

The presented framework allows a novel insight into the common understanding of M_0_ and T_1_ that are conventionally regarded as properties of the free pool only. The proposed approach makes explicit not only the existence of a $M_{0}^{\mathrm{CSMT}}$ and $T_{1}^{\mathrm{CSMT}}$ dependent on the single parameter $B_{1}^{\mathrm{rms}}$ (Figure 7), but also, as expressed in Equations (5) and (6), a clear dependence on the underlying MT properties of the multi‐pool system. This allows, for the first time, a clear bridge that makes single‐pool relaxometry consistent with the quantitative MT literature^10^, ^47^, ^48^ without the need for more complete modeling strategies featuring multiple pools of magnetization.^7^, ^8^, ^11^, ^12^, ^14^, ^34^ Furthermore, as evidenced by the data presented here (Figures 4 and 5), uncontrolled MT effects may explain the inconsistency in the current single‐pool relaxometry literature^28^ as the constituent measurements obtained from standard relaxometry methods (in which $B_{1}^{\mathrm{rms}}$ varies between acquisitions) would be inconsistent with one another, resulting in fitted parameters that are sensitive to incidental details of the measurement conditions. Depending on specific details of different manufacturers’ software, $B_{1}^{\mathrm{rms}}$ is expected to vary even for sequences with nominally equal parameters. This might explain the range of estimated values across difference studies even though nominally similar methods are used. The estimated parameters are only consistent when $B_{1}^{\mathrm{rms}}$ is held constant, which is possible via the proposed the CSMT framework. The observed T_2_ is shown to be both independent of the saturation conditions, if measurements with consistent $M_{0}^{\mathrm{CSMT}}$ and $T_{1}^{\mathrm{CSMT}}$ are obtained, and to agree with gold‐standard spin‐echo measurements^46^ as expected from our mathematical derivation. These results emphasize that single‐pool relaxometry measurements should be qualified, perhaps even defined, by the RF power used. It is also plausible that these compound parameters will be sensitive to tissue changes associated with pathology, because it is well‐established that diseases such as multiple sclerosis and Alzheimer's disease result in diffuse changes in MT.^49^, ^50^ This provides a motivation for further exploration of CSMT relaxometry as a quantitative radiological tool.

We note this methodology goes against the current trend of knowledge where a single, tissue‐specific, recovery time is sought that will be common between both SS and non‐SS gold‐standard inversion recovery methods for relaxometry.^27^ It is now well‐established^10^ that for a tissue characterized by the 2‐pool MT model, longitudinal relaxation exhibits a bi‐exponential response. Inversion recovery experiments with sufficiently long inversion delay times will measure the slower of the 2 rates, and this rate may be written as a composite of the underlying 2‐pool model parameters.

(11) $$R_{1}^{\mathrm{obs}}=\frac{1}{2}\left(R_{1}^{f}+\kappa^{f}+R_{1}^{m}+\kappa^{m}\right)-\frac{1}{2}\sqrt{{\left(R_{1}^{f}+\kappa^{f}+R_{1}^{m}+\kappa^{m}\right)}^{2}-4\left(R_{1}^{f}R_{1}^{m}+R_{1}^{f}\kappa^{m}+R_{1}^{m}\kappa^{f}\right)}\;=\frac{1}{T_{1}^{\mathrm{obs}}}$$

A corollary of the CSMT framework is that SS methods sample a different interaction of the MT tissue model; even in the limit of $B_{1}^{\mathrm{rms}}\to 0$, the 2 measurable recovery rates are not expected to agree (ie, $R_{1}^{\mathrm{obs}}\neq R_{1}^{\mathrm{csmt}}\left(B_{1}^{\mathrm{rms}}\to 0\right)$). As an example, using the same tissue parameters for WM as the numerical validation described above, we expect $R_{1}^{\mathrm{obs}}=1.09s^{-1}$ whereas $R_{1}^{\mathrm{csmt}}=1.25s^{-1}$.

Although well‐established as a reproducible measure, $R_{1}^{\mathrm{obs}}$ is also not immune to MT saturation‐related inaccuracies.^51^, ^52^ For example, in a multi‐slice experiment, the RF pulses acting on each slice will also act as off‐resonant saturation of the macromolecular pool for neighbors^16^ resulting in altered dynamics. An exploratory study^53^ demonstrated that multi‐slice acquisitions have a significant effect on the measured relaxation values in collagen samples with increasing concentrations. Another recent study showed that the type of inversion pulse used for 2D inversion recovery imaging will also affect the measured signal, because inversion pulses with different amounts of total energy lead to different amounts of macromolecular pool saturation.^52^ With this in mind, we explored adapting the CSMT framework for non‐SS methods such as MP2RAGE^23^ or MRF^5^ to stabilize the obtained measurements and bring a consensus with SS methodology. Although an experimental validation is outside the scope of this article, a mathematical derivation can be found in the Supporting Information where the well‐known bi‐exponential recovery of a 2‐pool MT system^10^, ^38^, ^51^, ^52^ can be adapted for the scenario of a constant $B_{1}^{\mathrm{rms}}$. For single‐slice or 3D imaging, this could be achieved experimentally by continuous application of off‐resonance saturation pulses during the sequence. From the derivation, it can be observed that full agreement (ie, equal measured recovery time at equivalent power $B_{1}^{\mathrm{rms}}$) between non‐SS and SS recovery can only be obtained in the limit of full saturation of the macromolecular pool ($B_{1}^{\mathrm{rms}}\to \infty$). Simulations based on literature WM 2‐pool MT parameters^34^ suggest good agreement (<10% difference) can be obtained when operating with $B_{1}^{\mathrm{rms}}$ at maximum safety limits for deposited power.

We further highlight $T_{1}^{\mathrm{CSMT}}$ dependency on $B_{1}^{\mathrm{rms}}$ in the in vivo experiment summarized in Figure 7. Here, a significant variation of the measured $T_{1}^{\mathrm{CSMT}}$ values is shown for both WM‐ and GM‐specific ROIs. The presented data show that $T_{1}^{\mathrm{CSMT}}$ variations can be mitigated by operating in a low SAR regime (eg, low FA and long T_R_) as there is limited $T_{1}^{\mathrm{CSMT}}$ variation between $B_{1}^{\mathrm{rms}}=0.2\mu T$ and $B_{1}^{\mathrm{rms}}=0.4\mu T$. Hence the proposed CSMT framework is expected to have a significant impact of T_2_ estimation through SS sequences. However, shorter repetition times and higher FAs are typically required,^20^, ^26^, ^29^ which naturally lead to higher values of $B_{1}^{\mathrm{rms}}$ and as a consequence, shorter $T_{1}^{\mathrm{CSMT}}$ values.

For SS methods, $B_{1}^{\mathrm{rms}}$ is easily controllable simply by adjusting TR, at the expense of acquisition time. However, such a naive approach would force sub‐optimal parameter choices in terms of overall estimation precision.^29^ The proposed CSMT via non‐selective MB excitation allows customization of the pulse energy giving full flexibility to design relaxometry protocols at fixed values of $B_{1}^{\mathrm{rms}}$. For slice‐selective applications, dedicated pulse design strategies need to be used where multiple FAs can be achieved at constant $B_{1}^{\mathrm{rms}}$. As an example, one can easily envision a simultaneous multi‐slice acquisition where several slices are obtained simultaneously with different FAs to ensure CSMT conditions while maintaining acquisition time efficiency. In Figure 7, it is demonstrated experimentally that the value of $B_{1}^{\mathrm{rms}}$ impacts the in vivo measured $T_{1}^{\mathrm{CSMT}}$. Although this appears to be an unfamiliar result at first, the range of obtainable relaxation times is in accordance with the panoply of relaxation times found throughout the literature, where relaxometry methods using only SPGR measures have reported WM T_1_ values between 801 ms and 1735 ms^28^ It is therefore suggested that, in complex samples, the applied $B_{1}^{\mathrm{rms}}$ must be reported, especially in measurements from multi‐center studies where absolute reported relaxation parameter values that characterize the measured object are sought. Perhaps even a standardized fixed power level should be agreed on by the community.

The proposed framework is valid as long as our initial assumption of CW‐equivalent saturation holds. To explore the limits of such assumption, a numerical simulation was performed where SS obtained for a bSSFP sequence is compared for both pulsed saturation and CW equivalent (non‐pulsed). This is highlighted in Supporting Information Figure S1 where good agreement (<1% error) in the measured signal is obtained between pulsed and CW‐equivalent simulations.

Transmit field corrections are necessary^54^, ^55^ to correctly characterize the rotation induced in the free pool, however, it is currently not feasible to account for the effect of corresponding variation of $B_{1}^{\mathrm{rms}}$ on the saturation achieved in the macromolecular pool. From Equations (5) and (6), it is expected there will be a residual spatial variation in the final estimated $M_{0}^{\mathrm{CSMT}}$ and $T_{1}^{\mathrm{CSMT}}$ maps because of this effect, although not in the determination of T_2_. This effect is minimized at low field strength (≤1.5 T) where spatial variations of transmit field are less—at high field strengths (eg, 7.0 T), alternative solutions are needed, and this is a focus for future research. From the measured spatial variation in RF amplitude of the experiment reported in Figure 5, numerical simulations based on previously reported quantitative MT parameters^34^ are predicted to induce a variation in $T_{1}^{\mathrm{CSMT}}$ of 16% at 3 T.

## 6 CONCLUSION

This article presents a framework that enables consistent results to be obtained from single‐pool relaxometry in the presence of confounding magnetization transfer effects, which are ubiquitous in human tissues. This has the potential to reduce the currently observed inconsistency between relaxometry methods without requiring any more data than are currently acquired. The approach is compatible with a diverse range of existing high resolution in vivo relaxometry methods, and the data presented provides the first evidence that it can lead to stable and consistent results. Here, we made use of dedicated RF pulses that allow flexible balance between off‐ and on‐resonance energy to obtain at fixed $B_{1}^{\mathrm{rms}}$ in non‐selective SS sequences. However, this is just an optimized methodology for brain imaging, and slice‐selective approaches with CSMT conditions can be achieved by more conventional methods with separate off‐resonance pulses, provided $B_{1}^{\mathrm{rms}}\;$ is held constant for all measurements and is quoted as a key parameter in any reported results. In this work, we have operated at an arbitrary value of 2.06 µT, however, because $B_{1}^{\mathrm{rms}}$ can be simply determined by time integration of the RF pulses used in acquisition sequences, an agreed value can be achieved while still allowing latitude for differences in implementation details that may be system or manufacturer specific. A central consequence of the CSMT approach is that the measured proton density and longitudinal relaxation time both depend on $B_{1}^{\mathrm{rms}}\;$ as both are measures of the underlying multi‐compartment nature of the tissue concerned. This is a clear deviation from a frequent current interpretation of M_0_ as the magnetization of the visible pool when fully relaxed and T_1_ as a spin‐lattice relaxation time that excludes magnetization transfer. This change may take some getting used to, particularly as the absolute values of the quantities concerned can be quite different when there are substantial MT processes operating within tissues. The use of $M_{0}^{\mathrm{CSMT}}\left(B_{1}^{\mathrm{rms}}\right)$, $T_{1}^{\mathrm{CSMT}}\left(B_{1}^{\mathrm{rms}}\right)$, and T_2_ as quantitative measures to efficiently characterize tissue properties in health and disease remains to be proven in large scale studies, but the wealth of literature that explicitly accounts for MT supports their usage.

## ACKNOWLEDGMENT

This work received funding from the European Research Council under the European Union’s Seventh Framework Programme (FP7/20072013/ERC grant agreement no. [319456] dHCP project) and MRC strategic grant [MR/K006355/1]. The research was supported by the National Institute for Health Research (NIHR) Biomedical Research Centre based at Guy's and St Thomas' NHS Foundation Trust and King’s College London. The views expressed are those of the author(s) and not necessarily those of the NHS, the NIHR or the Department of Health. This work was additionally supported by the Wellcome/EPSRC Centre for Medical Engineering at King’s College London [WT 203148/Z/16/Z].

## Supporting information


**FIGURE S1** Comparison of measured signal (top), free pool longitudinal magnetization (middle) and macromolecular longitudinal magnetization (bottom) between pulsed and non‐pulsed bSSFP steady‐state for a fixed free pool flip angle of 68°.

## APPENDIX A

### 2‐pool MT as single pool.

Here, we described the algebraic manipulations required to identify the single‐pool behavior of a 2‐pool MT system when in steady state. Building from Equation (4), it can be rearranged to make its subject $M_{z}^{m}$:

(A1) $$M_{z}^{m}=-\frac{\kappa^{m}\frac{dM_{z}^{m}}{\mathrm{dt}}}{R_{1}^{m}+\kappa^{m}+\overset{¯}{W}}+\frac{\kappa^{f}}{R_{1}^{m}+\kappa^{m}+\overset{¯}{W}}M_{z}^{f}+\frac{R_{1}^{m}M_{0}^{m}}{R_{1}^{m}+\kappa^{m}+\overset{¯}{W}}$$

that can be substituted into Equation (3) such that, under free‐precession with constant saturation, it becomes.

(A2) $$\frac{dM_{z}^{f}}{\mathrm{dt}}=R_{1}^{f}\left(M_{0}^{f}-M_{z}^{f}\right)-\kappa^{f}M_{z}^{f}+\kappa^{m}\left[-\frac{\kappa^{r}\frac{dM_{z}^{m}}{\mathrm{dt}}}{R_{1}^{m}+\kappa^{m}+\leq \overset{¯}{W}>}+\frac{\kappa^{f}}{R_{1}^{m}+\kappa^{m}+\overset{¯}{W}}M_{z}^{f}+\frac{R_{1}^{m}M_{0}^{m}}{R_{1}^{m}+\kappa^{m}+\overset{¯}{W}}\right]$$

Rearranging the terms, we obtain.

(A3) $$\frac{dM_{z}^{f}}{\mathrm{dt}}=-\left[R_{1}^{f}+\kappa^{f}\left(1-\frac{\kappa^{m}}{R_{1}^{m}+\kappa^{m}+\overset{¯}{W}}\right)\right]M_{z}^{f}-\frac{\kappa^{m}\frac{dM_{z}^{m}}{\mathrm{dt}}}{R_{1}^{m}+\kappa^{m}+\overset{¯}{W}}+\frac{R_{1}^{m}M_{0}^{m}}{R_{1}^{m}+\kappa^{m}+\overset{¯}{W}}+R_{1}^{f}M_{0}^{f}$$

We note that Equation (A3) is similar in form to the single‐pool recovery $\frac{dM_{z}^{f}}{\mathrm{dt}}=-R_{1}M_{z}+R_{1}M_{0}$. Hence, it can be expressed as

(A4) $$\frac{dM_{z}^{f}}{\mathrm{dt}}=-R_{1}^{\mathrm{CSMT}}M_{z}^{f}+R_{1}^{\mathrm{CSMT}}M_{0}^{\mathrm{CSMT}}$$

## APPENDIX B

### Pulsed steady‐state solution of a 2‐pool system.

The magnetization evolution of MT systems, including the free‐pool transverse magnetization and assuming the continuous wave approximation, is given by the coupled Bloch equations:

(B1) $$\frac{d}{\mathrm{dt}}\left[\begin{array}{c} M_{x}^{f}\\ M_{y}^{f}\\ M_{z}^{f}\\ M_{z}^{r} \end{array}\right]=\left[\begin{array}{cccc} -R_{2} & \Delta \omega_{0} & 0 & 0\\ -\Delta \omega_{0} & -R_{2} & 0 & 0\\ 0 & 0 & -R_{1}^{f}-\kappa^{f} & \kappa^{r}\\ 0 & 0 & \kappa^{f} & -R_{1}^{r}-\kappa^{r} \end{array}\right]\left[\begin{array}{c} M_{x}^{f}\\ M_{y}^{f}\\ M_{z}^{f}\\ M_{z}^{r} \end{array}\right]+\left[\begin{array}{c} 0\\ 0\\ R_{1}^{f}M_{z}^{f}\\ R_{1}^{r}M_{z}^{r} \end{array}\right]=\mathrm{AM}+B$$

This system of coupled differential equations has the formal solution given by:

(B2) $$\left[\begin{array}{c} M_{x}^{f}\left(t\right)\\ M_{y}^{f}\left(t\right)\\ M_{z}^{f}\left(t\right)\\ M_{z}^{r}\left(t\right) \end{array}\right]=e^{\mathrm{At}}\left(M_{t=0}+A^{-1}B\right)-A^{-1}B$$

As we approximate the saturation effect of the RF pulse as a continuous wave equivalent, its effect is well‐described by the rotation operator:

$$R=\left[\begin{array}{cccc} 1 & 0 & 0 & 0\\ 0 & \cos\left(\alpha^{f}\right) & \sin\left(\alpha^{f}\right) & 0\\ 0 & -\sin\left(\alpha^{f}\right) & \cos\left(\alpha^{f}\right) & 0\\ 0 & 0 & 0 & e^{-\leq W>\tau_{\mathrm{RF}}} \end{array}\right]$$

Following the notation where the magnetization after the $n^{\mathrm{th}}$ RF pulse is given in terms of the magnetization before that pulse by $M_{n}^{+}=RM_{n}^{-}$, and identifying that in the steady state $M_{n+1}^{+}=M_{n}^{+}$, a formal solution can be derived as follows:

(B4) $$M_{n+1}^{-}=e^{\mathrm{At}}\left(M_{n}^{+}+A^{-1}B\right)-A^{-1}B=e^{\mathrm{At}}M_{n}^{+}+\left(e^{\mathrm{At}}-I\right)A^{-1}B$$

(B5) $$M_{n+1}^{+}=RM_{n+1}^{-}=Re^{\mathrm{At}}M_{n}^{+}+R\left(e^{\mathrm{At}}-I\right)A^{-1}B$$

(B6) $$M^{\mathrm{SS}}=Re^{\mathrm{At}}M^{\mathrm{SS}}+R\left(e^{\mathrm{At}}-I\right)A^{-1}B$$

(B7) $$M^{\mathrm{SS}}-Re^{\mathrm{At}}M^{\mathrm{SS}}=R\left(e^{\mathrm{At}}-I\right)A^{-1}B$$

(B8) $$\left(I-Re^{\mathrm{At}}\right)M^{\mathrm{SS}}=R\left(e^{\mathrm{At}}-I\right)A^{-1}B$$

(B9) $$M^{\mathrm{SS}}={\left(I-Re^{\mathrm{At}}\right)}^{-1}R\left(e^{\mathrm{At}}-I\right)A^{-1}B$$

Equations (B1)–(B9) summarize the formal derivation of the 2‐pool bSSFP signal. If an SPGR signal is sought, spoiling of the magnetization needs to be considered before the rotation of the free pool. With this, the same formalism can be used with the simple substitution R→RSR \to RS, where the operator S is given by:

(B10) $$S=\left[\begin{array}{cccc} 0 & 0 & 0 & 0\\ 0 & 0 & 0 & 0\\ 0 & 0 & 1 & 0\\ 0 & 0 & 0 & 1 \end{array}\right]$$

and Equation (B9) then becomes:

(B11) $$M^{\mathrm{SS}}={\left(I-RSe^{\mathrm{At}}\right)}^{-1}RS\left(e^{\mathrm{At}}-I\right)A^{-1}B$$
