# Supplementary material for: Fast quantitative MRI using controlled saturation magnetization transfer
Source: Magn Reson Med. 2018 Sep 14;81(2):907–20. doi: 10.1002/mrm.27442 (PMC6492254; doi:10.1002/mrm.27442)
Supplement: Supplementary file 1 — FIGURE S1 Comparison of measured signal (top), free pool longitudinal magnetization (middle) and macromolecular longitudinal magnetization (bottom) between pulsed and non‐pulsed bSSFP steady‐state for a fixed free pool flip angle of 68°. [file MRM-81-907-s001.docx]

Fast quantitative MRI using Controlled Saturation of Magnetization Transfer – Supplementary Information

# Continuous wave equivalent versus Pulses Saturation

A key assumption of the proposed CSMT framework is that for short TR SS sequences, the response time of the bound pool longitudinal magnetization is slow varying and hence, once a steady-state has been formed it can be approximated by an average saturation rate $<\overline{W}>$. In order to probe the limits of this assumption the following numerical experiment was generated: a bSSFP steady-state signal was modelled via 1) pulsed saturation at each excitation and 2) with the proposed CW equivalent (non-pulsed). This is done for a fixed ${68}^{\circ}$ flip angle at different RF-energies and with representative tissue parameters extracted from (1): $M_{0}^{f}=1;M_{0}^{m}=0.157;k^{f}=4.45 s^{-1};k^{m}=28.34 s^{-1};R_{1}^{f}=1.1 s^{-1};R_{1}^{m}=1 s^{-1};R_{2}^{f}=12.3 s^{-1};G=14 \mu s$; The percentage deviation (color scale) of the obtained signal ($M_{xy}$), free pool longitudinal magnetization ($M_{z}^{f}$) and macromolecular pool longitudinal Magnetization ($M_{z}^{m}$) using the CW approach relative to the pulsed approach is shown in Figure SI1.


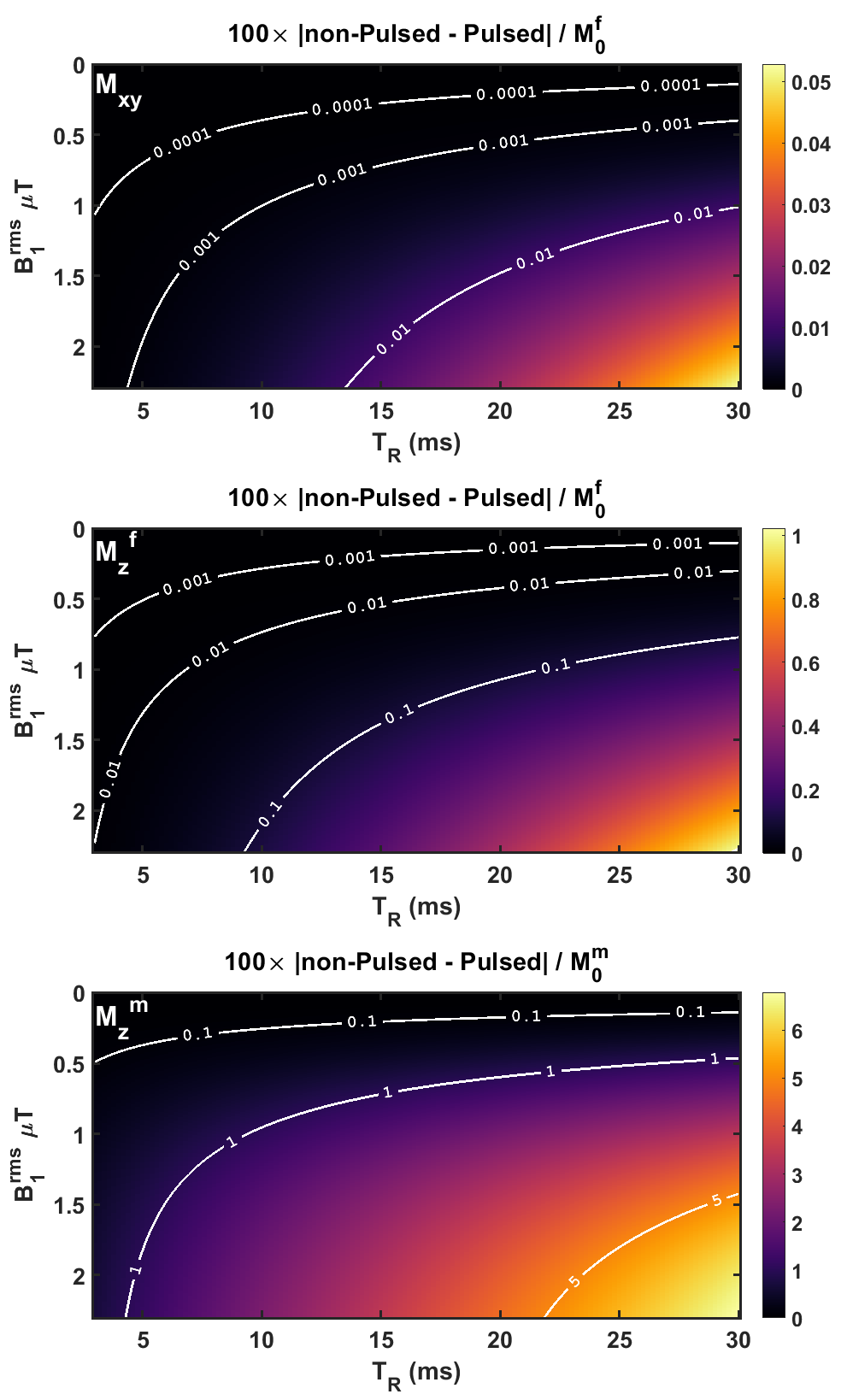


Supporting Information Figure S1 - Comparison of measured signal (top), free pool longitudinal magnetization (middle) and macromolecular longitudinal magnetization (bottom) between pulsed and non-pulsed bSSFP steady-state for a fixed free pool flip angle of ${68}^{\circ}$.

From Supporting Information Figure S1 it can be seen that there is an error in the macromolecular longitudinal magnetization using the CW approximation. Nevertheless, this error is expected to be <2% for TR<10ms at all reasonable $B_{1}^{rms}$ explored at 3T. Furthermore, both $M_{xy}$ and $M_{z}^{f}$seem to be more robust to the proposed approximation as the errors obtained are expected to be <1% for all the regimes explored.

# Continuous wave two-pool MT with non-SS methods

Following the derivation of two-pool MT magnetization evolution presented in 1996 by G. Bruce Pike(2):

$$\left[ Eq SI1.1 \right]\frac{d}{dt}\left[ \begin{matrix} M_{z}^{f} \\ M_{z}^{m} \end{matrix} \right]=\left[ \begin{matrix} -R_{1}^{f}-\kappa^{f} & \kappa^{m} \\ \kappa^{f} & -R_{1}^{m}-\kappa^{m} \end{matrix} \right]\left[ \begin{matrix} M_{z}^{f} \\ M_{z}^{m} \end{matrix} \right]+\left[ \begin{matrix} R_{1}^{f}M_{0}^{f} \\ R_{1}^{m}M_{0}^{m} \end{matrix} \right]=A\mathbf{M}+B$$

A solution to the system of coupled differential equations is of the form(2):

$$[Eq SI1.2]M=C_{+}\left[ \begin{matrix} v_{\lambda_{+}}^{f} \\ v_{\lambda_{+}}^{r} \end{matrix} \right]e^{\lambda_{+}t}+C_{-}\left[ \begin{matrix} v_{\lambda_{-}}^{f} \\ v_{\lambda_{-}}^{r} \end{matrix} \right]e^{\lambda_{-}t}-A^{-1}B$$

where $\lambda_{+}$and$\lambda_{-}$ are the eigenvalues of the system typically described as(2,3):

$$\left[ Eq SI1.3 \right]\lambda_{\pm}=\frac{1}{2}\left( R_{1}^{f}+\kappa^{f}+R_{1}^{m}+\kappa^{m} \right)\pm\frac{1}{2}\sqrt{\left( R_{1}^{f}+\kappa^{f}+R_{1}^{m}+\kappa^{m} \right)^{2}-4\left( R_{1}^{f}R_{1}^{m}+R_{1}^{f}\kappa^{m}+R_{1}^{m}\kappa^{f} \right)}$$

Note that the slow recovery eigenvalue $\lambda_{-}$ is also commonly referred to as $R_{1}^{obs}=1/T_{1}^{obs}$ as it is the term measured from gold-standard IR experiments(4,5). Under the approximation of continuous saturation of the macromolecular pool of protons, the eigenvalues of the system include a term proportional to the average saturation rate $<\overline{W}>=\pi\left( {\gamma B}_{1}^{rms} \right)^{2}G$:

$$\left[ Eq SI1.4 \right]\lambda_{\pm}^{CSMT}=\frac{1}{2}\left( R_{1}^{f}+\kappa^{f}+R_{1}^{m}+\kappa^{m}+<\overline{W}> \right)\pm\frac{1}{2}\sqrt{\left( R_{1}^{f}+\kappa^{f}+R_{1}^{m}+\kappa^{m}+<\overline{W}> \right)^{2}-4\left( R_{1}^{f}R_{1}^{m}+R_{1}^{f}\kappa^{m}+R_{1}^{m}\kappa^{f}+\left( R_{1}^{f}+\kappa^{f} \right)<\overline{W}> \right)}$$

For ease of representation we define the variables:

$$\left[ Eq SI1.4 \right]b=R_{1}^{f}+\kappa^{f}+R_{1}^{m}+\kappa^{m}+<\overline{W}>$$

$$\left[ Eq SI1.5 \right]c=R_{1}^{f}R_{1}^{m}+R_{1}^{f}\kappa^{m}+R_{1}^{m}\kappa^{f}+\left( R_{1}^{f}+\kappa^{f} \right)<\overline{W}>$$

Such that Equation SI1.4 simplifies to:

$$\left[ Eq SI1.6 \right]\lambda_{\pm}^{CSMT}=\frac{1}{2}b\pm\frac{1}{2}\sqrt{b^{2}-4c}=\frac{1}{2}b\pm\frac{1}{2}b\sqrt{1-\frac{4c}{b^{2}}}$$

Assuming the absorption lineshape is constant(1,6) at $G=1.4\times{10}^{-5}s$ and using previously reported 2-pool model values(1), as a first order approximation, $\frac{4c}{b^{2}}<1$. Expansion of Equation $\mathrm{SI}$1.6 can therefore be obtained:

$$\left[ Eq SI1.7 \right]\lambda_{\pm}^{CSMT}\approx\frac{1}{2}b\pm\frac{1}{2}b\left( 1-\frac{1}{2}\frac{4c}{b^{2}} \right)=\frac{1}{2}b\pm\left( \frac{1}{2}b-\frac{c}{b} \right)$$

and therefore, the fast recovery$\lambda_{+}$ and slow recovery $\lambda_{-}$ terms can be easily identified as:

$$\left[ Eq SI1.8 \right]\lambda_{+}^{CSMT}\approx\frac{1}{2}b+\frac{1}{2}b-\frac{c}{b}=b-\frac{c}{b}=b\left( 1-\frac{c}{b^{2}} \right)$$

$$\left[ Eq SI1.9 \right]\lambda_{-}^{CSMT}\approx\frac{1}{2}b-\frac{1}{2}b+\frac{c}{b}=\frac{c}{b}$$

In the extreme limit where $B_{1}^{rms}\to\infty$, then $<\overline{W}>$term dominates the recovery ($b\approx<\overline{W}>$ and $c\approx\left( R_{1}^{f}+\kappa^{f} \right)<\overline{W}>$) and the fully saturated solution previous reported(2) can be recovered from Equations $SI1$.8 and $SI1$.9:

$$\left[ Eq SI1.10 \right]\lambda_{+}^{CSMT}\left( B_{1}^{rms}\to\infty\right)={\pi\left( \gamma b_{1}^{rms} \right)}^{2}G\left( 1-\frac{\left( R_{1}^{f}+\kappa^{f} \right)<\overline{W}>}{\left( <\overline{W}> \right)^{2}} \right)=<\overline{W}>-\left( R_{1}^{f}+\kappa^{f} \right)$$

$$\left[ Eq SI1.11 \right]\lambda_{-}^{CSMT}\left( B_{1}^{rms}\to\infty\right)=\frac{\left( R_{1}^{f}+\kappa^{f} \right)<\overline{W}>}{<\overline{W}>}=R_{1}^{f}+\kappa^{f}=R_{1}^{CSMT}(B_{1}^{rms}\to\infty)$$

# References

1. Gloor M, Scheffler K, Bieri O. Quantitative magnetization transfer imaging using balanced SSFP. Magn. Reson. Med. [Internet] 2008;60:691–700. doi: 10.1002/mrm.21705.

2. Pike GB. Pulsed magnetization transfer contrast in gradient echo imaging: a two-pool analytic description of signal response. Magn. Reson. Med. 1996;36:95–103. doi: 10.1002/mrm.1910360117.

3. Calucci L, Forte C. Proton longitudinal relaxation coupling in dynamically heterogeneous soft systems. Prog. Nucl. Magn. Reson. Spectrosc. [Internet] 2009;55:296–323. doi: 10.1016/j.pnmrs.2009.06.003.

4. van Gelderen P, Jiang X, Duyn JH. Effects of magnetization transfer on T1contrast in human brain white matter. Neuroimage [Internet] 2016;128:85–95. doi: 10.1016/j.neuroimage.2015.12.032.

5. Rioux J a., Levesque IR, Rutt BK. Biexponential longitudinal relaxation in white matter: Characterization and impact on T _1_ mapping with IR-FSE and MP2RAGE. Magn. Reson. Med. [Internet] 2015;0:n/a-n/a. doi: 10.1002/mrm.25729.

6. Gloor M, Scheffler K, Bieri O. Nonbalanced SSFP-based quantitative magnetization transfer imaging. Magn. Reson. Med. 2010;64:149–156. doi: 10.1002/mrm.22331.
